# Stability of matrix metalloproteinase-9 as biological marker in colorectal cancer

**DOI:** 10.1007/s12032-018-1109-4

**Published:** 2018-03-09

**Authors:** Andreas Jonsson, Claes Hjalmarsson, Peter Falk, Marie-Lois Ivarsson

**Affiliations:** 1 Hallands Hospital Varberg, Region Halland, 432 37 Varberg, Sweden; 2 Department of Surgery, Sahlgrenska University Hopital, Göteborg, Sweden; 3 Fibrinolysis Laboratory, Department of Surgery, Institute of Clinical Sciences, Sahlgrenska Academy, University of Gothenburg, 416 85 Göteborg, Sweden

**Keywords:** Colorectal cancer, MMPs, MMP-9, Prognostic marker

## Abstract

Matrix metalloproteinases (MMPs) are believed to be of importance in the growth and spread of colorectal cancer (CRC). MMP-9 level has been suggested as a biological predictor of prognosis in CRC as well as in other types of cancer such as breast and cervical cancer. The purpose of this study was to investigate the stability over time of MMP-9 in cryopreserved plasma, colorectal tumor tissue extract and macroscopically tumor-free colon mucosa tissue extract samples. Plasma and tissue samples were taken from patients at primary CRC surgery and analyzed for MMP-9. Aliquots of samples from the same patients were stored at – 80 °C pending analysis. These aliquots were analyzed using identical methods after storage periods of nine (plasma) and twelve (tissue) years. No significant difference in plasma MMP-9 concentration was seen between baseline samples and those after 9 years of cryopreservation (median values 9.9 and 9.7 ng/mL, respectively; *p* > 0.05). MMP-9 levels in the tumor-free tissue extracts had increased to baseline (median values 7.1 and 8.1 ng/mL, respectively; *p* < 0.01). MMP-9 levels in the tumor tissue extracts had also increased significantly (median values 89.9 and 133.5 ng/mL, respectively; *p* < 0.01). We have demonstrated that MMP-9 levels in frozen citrated plasma are stable if stored at − 80 °C, whereas MMP-9 levels in extracts from tumor tissue and tumor-free intestinal mucosa appear to increase with time. We conclude that MMP-9 levels in cryopreserved plasma may be considered stable over time and are thus suitable for comparison purposes in consecutive series.

## Introduction

The matrix metalloproteinases (MMPs) belong to a family of zinc- and calcium-dependent proteolytic enzymes that are important in the degradation of extracellular matrix (ECM) in many types of cancer [1–4]. More than 25 MMPs have been identified [5, 6], and their regulation is controlled by several mechanisms including transcription, activation, and inhibition. Tumor cells and surrounding stromal cells are all able to synthesize MMPs [5].

MMP-9 is one of the key proteolytic enzymes in the breakdown and reconstruction of ECM in colorectal cancer (CRC) invasion and metastasis. MMP-9 regulates the microenvironment around the tumor and increases the concentration of vascular endothelial growth factor (VEGF) that regulates angiogenesis [7]. MMP-9 is also active in the formation of early metastatic niches [8]. In preclinical models, selective MMP-9 inhibitors have been shown to decrease tumor growth and the incidence of metastases in colorectal cancer and also induce cancer cell apoptosis in pancreatic cancer [9, 10].

Several studies have demonstrated elevated levels of MMP-9 in the tumor tissue and plasma of patients with CRC [11, 12], and MMP-9 level has been suggested as a biological predictor of prognosis in CRC, as well as in other types of cancer such as breast and cervical cancer [13, 14]. Furthermore, in patients with CRC, MMP-9 levels in adjacent tumor-free mucosa are elevated and this could be used as a predictor of 5-year relative survival in colorectal cancer [15]. Also fecal MMP-9 measurement has recently come up as a promising marker for CRC [16].

Studies on cancer-related outcome often use consecutive case series where blood and tissue samples are collected and frozen pending analysis. To minimize inter-assay variation, samples are often thawed and analyzed in batches. In this respect, there are concerns regarding the stability of MMP levels in plasma and tissue samples that are cryopreserved for long periods of time [17]. Previous studies have shown that inappropriate handling of specimens may lead to degradation of biomarkers in cancer tissue [18]. This is of greatest concern in longitudinal trials investigating disease progression. It is also important how blood is handled prior to MMP analysis, and whether it is serum or plasma that is used [19–21].

To the best of our knowledge, no data have been published regarding the long-term cryostability of MMP-9 in plasma, or in centrifugal extracts of tumor-free intestinal mucosa or CRC tumor tissue, where samples have been stored for periods longer than 4 years [22–24].

The aim of this study was to investigate the long-term stability of MMP-9 levels in cryopreserved citrated plasma, and centrifugal extracts of tumor tissue and tumor-free intestinal mucosa samples from patients with CRC, thereby exploring the possibility to use cryopreserved samples in future research on consecutive case series.

## Materials and methods

### Patients and sample preparation

During the period February 1999–2005, blood samples, tumor tissue and biopsies from macroscopically tumor-free mucosa were obtained from patients undergoing surgery for colorectal cancer at the Department of Surgery, Sahlgrenska/Ostra Hospital, Gothenburg, Sweden.

Blood samples were collected in citrate tubes and then centrifuged within 5 min at 10000*g* for 10 min at 20 °C. The supernatant (citrated plasma) samples were then frozen in several aliquots at − 80 °C pending further analysis.

Tissues samples approximately 1 cm^2^ in area were taken from the area of the tumor, and from tumor-free mucosa approximately 10 cm from the tumor. Tissue samples were immediately frozen in liquid nitrogen in the operating theater and stored at − 80 °C pending homogenization and centrifugation (see below).

### Tissue extract preparation

At the laboratory, tissue samples were thawed, weighed and then homogenized in 1 ml PBS buffer with 0.01% Triton X-100 per 40 mg tissue, and centrifuged at 10000*g* for 10 min. The supernatant from each of the homogenized tissue sample was then extracted and frozen in several aliquots at − 80 °C pending analysis.

### MMP-9 level measurement and data collection

Aliquots from both plasma and tissue supernatant samples were analyzed in batches in conjunction with the surgical procedure (baseline), while aliquots from further plasma and tissue supernatant samples were kept frozen at − 80 °C pending later analysis. After 9 years, plasma sample aliquots were thawed and analyzed for MMP-9, and corresponding aliquots of tissue supernatant samples from tumor and tumor-free tissue were analyzed 3 years later. MMP-9 levels were then compared to baseline aliquot levels from corresponding patients.

All samples were measured in duplicate, and the level of MMP-9 was calculated from the standard curve supplied by the manufacturer. Commercially available ELISA kits from Amersham GE Healthcare/VWR (Stockholm, Sweden) were used (kit: RPN2614). The intra-and inter-assay coefficients of variability were 4.9–5.5 and 8.1–9.8%, respectively, and the lower detection limit was 0.6 ng/mL. Measurements were based on a 96-well ELISA plate system using optical density at 450 nm for determination of concentrations in a multiwell-based plate reader connected to computer software (V-max and SoftMax Pro, Molecular Devices, USA). Sample concentrations were calculated from absorbance using a standard curve and internal controls with known concentrations.

The MMP-9 assay technique (RPN2614/Amersham GE Healthcare/VWR) used for the sample analyses at 9 and 12 years was equivalent to the assay used at baseline and performed in an identical manner using the same chemicals and methodology.

### Statistical analysis

Aliquots from the tumor and tumor-free mucosa extracts, and plasma samples were divided into two groups: baseline and cryopreserved. These were analyzed for MMP-9 at baseline, 9 years (plasma) and 12 years (tissue extracts), respectively, and the results presented as median and interquartile range. Since the data were not normally distributed, the Wilcoxon signed rank test was used to compare paired samples at baseline and at 9 (plasma) and 12 (extract) years. Correlations between each baseline and later analyses were estimated using the Spearman rank correlation test. Bland–Altman curves were plotted to measure agreement between the baseline and later measurements. In the plot, a confidence interval of 95% of the mean difference of recovery rate was used [25].

All tests were two-sided, and a *p* value of < 0.05 was considered significant. All calculations were carried out using IBM SPSS Statistics for Macintosh (Ver. 22.0, IBM Corp, Armonk, NY, USA).

## Results

At the time of analysis of the cryopreserved aliquots, the plasma samples had been stored frozen for 9 years and the tissue samples for 12 years. The baseline analyses of plasma and tissue sample aliquots were all performed within 24 months of harvesting from the patients in the operating theater.

In total, 36 samples from plasma, tumor-free tissue and tumor tissue were analyzed. Plasma and tissue extracts were co-analyzed at baseline and after cryopreservation.

**Fig. 1:**
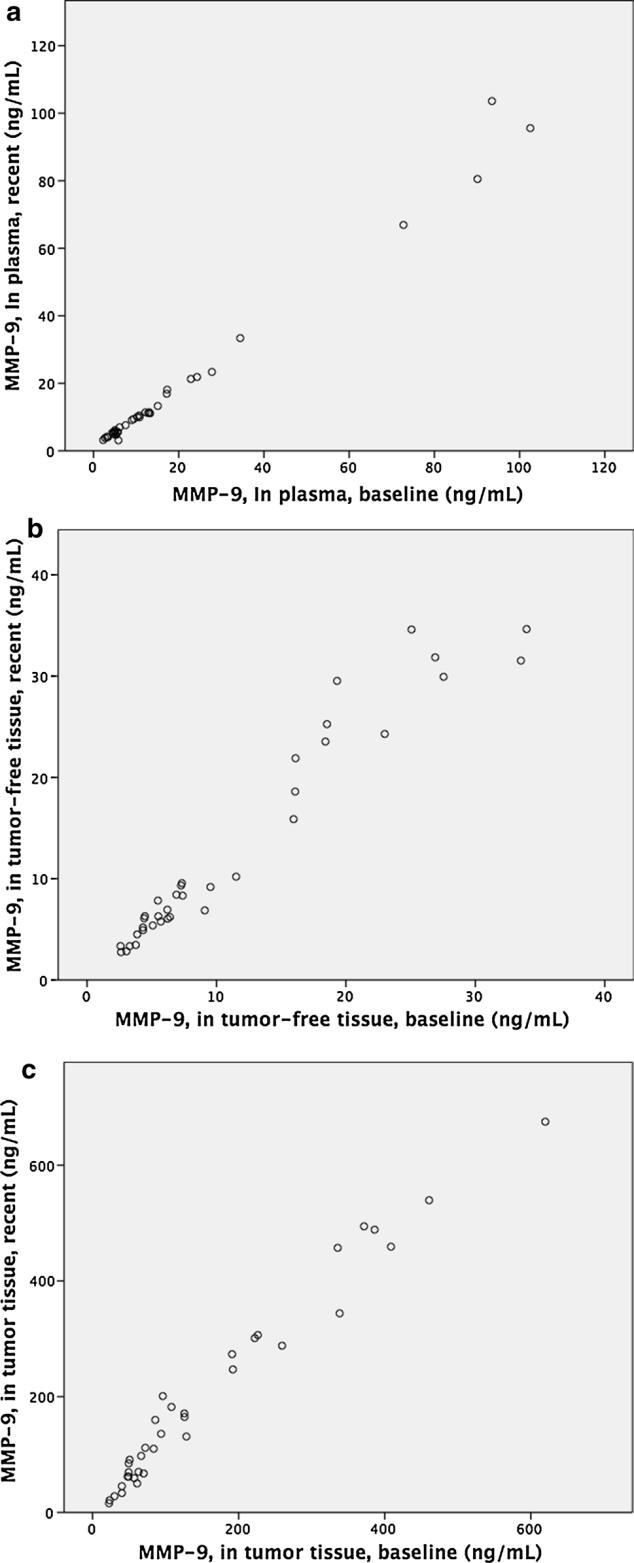
Correlations between baseline, precryopreservation, and recent, postcryopreservation, concentrations of matrix metalloproteinases (MMP). **a** Correlation between MMP-9 analyzed in citrate plasma samples before and after cryopreservation. **b** Correlation between the concentration of MMP-9 in baseline samples and cryopreserved samples of tumor-free intestinal tissue. **c** Correlation between concentration of MMP-9 in tumor tissue at baseline and after cryopreservation

In the plasma analyses, baseline MMP-9 levels had a median concentration of 9.9 ng/mL, interquartile range (IQR) 12.2 and range 2.28–102.5 ng/mL. After 9 years cryopreservation, the median level was 9.7 ng/mL, IQR 12.4 and range 3.1–103.6 ng/mL, correlating well with the baseline value (*ρ* = 0.96 (*p* < 0.01)) (Fig. 1). According to the Wilcoxon signed rank test, there was no significant difference between baseline and postcryopreservation MMP-9 levels (*p* > 0.05). The recovery rate was approximately 100% and did not differ more than the intra- and inter-assay coefficient of variability provided by the manufacturer of the assay. This is also shown in the Bland–Altman plot where values are presented near the zero mark.

In the tumor-free intestinal tissue extract analyses, baseline MMP-9 levels had a median concentration of 7.1 ng/mL (13.4), range 2.6–34.0 ng/mL. As with the plasma levels, the tumor-free intestinal tissue extract MMP-9 levels remained similar after 12 years of cryopreservation with a median concentration of 8.1 ng/mL (17.7), range 2.8–34.6 ng/mL, correlating well with baseline levels [*ρ* = 0.97 (*p* < 0.01)], see Fig. 1. However, when applying the paired Wilcoxon signed rank test, the difference between baseline and postcryopreservation levels was significant (*n* = 36, *p* < 0.01).

In the tumor tissue extract analyses, baseline MMP-9 levels had a median concentration of 89.9 ng/mL (175.4) range 22.6–619.9 ng/mL. Even here there was a good correlation between baseline and postcryopreservation levels, the latter showing a median of 133.5 ng/mL (234.5) range 15.9–675.1 ng/mL, [*ρ* = 0.97 (*p* < 0.01)]; see Fig. 1. As with the tumor-free tissue extract, however, when baseline and postcryopreservation values were compared pair-wise using the Wilcoxon signed rank test, a significant difference between the paired samples was revealed (*p* < 0.01).

**Fig. 2:**
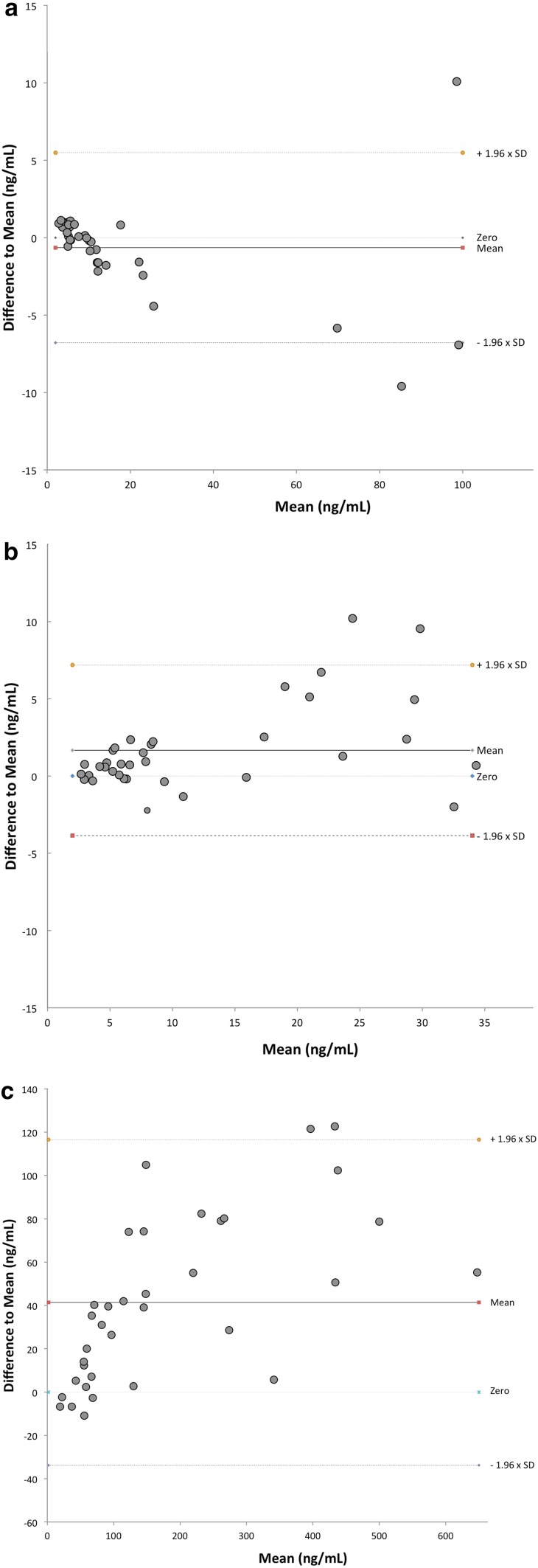
Bland–Altman plot showing the difference to mean for each analysis. Dots that are between upper and lower dotted lines represent values within the 95% confidence interval. **a** Bland–Altman plot of cryopreserved plasma samples. **b** Cryopreserved healthy tissue samples and **c** cryopreserved tumor tissue samples

The tissue sample analysis according to the Wilcoxon signed rank test is reflected in the Bland–Altman plots where there is a wider spread in the difference to the mean. This implies that the concentration of MMP-9 in tissue samples increases during cryopreservation; see Fig. 2. All medians and ranges are reported in detail in Table 1.

**Table 1:** Medians, interquartile ranges and Spearman rho of MMP levels at baseline and present

	Baseline analysis	Present analysis	*p* value	*ρ* (*p* value)
MMP-9 P [ng/mL (IQR)]	9.9 (12.2)	9.7 (12.4)	0.13	0.96 (< 0.01)
MMP-9 HIT[ng/mL (IQR)]	7.1 (13.4)	8.1 (17.7)	< 0.01	0.97 (< 0.01)
MMP-9 TT [ng/mL (IQR)]	89.9 (175.4)	133.5 (234.5)	< 0.01	0.97 (< 0.01)

*MMP* matrix metalloproteinases, *IQR* interquartile ranges, *P* Plasma, *TT* tumor tissue, *HIT* healthy intestinal tissue

## Discussion

Using standardized sampling conditions, we have studied the cryostability of MMP-9 in plasma as well in tissue extracts in a real-time setting. The samples were kept in storage for periods longer than previously investigated: up to 144 months compared to 43 months reported previously [22–24]. Using methods described previously, the present study shows that MMP-9 levels in frozen citrated plasma remain stable over a long period of time (Wilcoxon signed rank test *p* > 0.05). Comparable comparisons regarding tumor tissue extract and tumor-free mucosa tissue extract using Wilcoxon signed rank test revealed significant differences between baseline and after cryopreservation despite the Spearman test showing the concentrations to be well-correlated. The Bland–Altman test for agreement between the groups showed that the measurement values were mainly to be found in our 95% CI construct. Whatever, it seems clear that in the setting of this study, MMP-9 levels in tissue extracts from tumor and healthy mucosa increased over the period of cryopreservation.

There are other ways to determine the long-term stability of biomarkers. One way of assessing the stability of a biomarker in biological samples stored over long periods of time is to apply the Arrhenius equation, a so-called accelerated stability test. The equation can incorporate various types of stress applied to the sample at hand, such as elevated temperature, humidity, radiation. The equation may be used to describe how a chemical reaction depends on temperature, and the cryostability of MMPs has been investigated using this method [26].

Since the equation depends on assumptions regarding the properties of the biomarker at hand, there is always the risk of error. Reports on the real-time stability of biomarkers, including MMP-9, in samples stored for up to a decade are rare, presumably because of the lack of samples and data from the past, as well as modifications in detection kits provided by the industry. Real-time stability experiments are superior to mathematic models since they more accurately represent the clinical setting.

Our results showed that MMP-9 levels in frozen citrated plasma are very stable, the recovery rate is near 100%, the paired test revealed no significant difference, and the correlation was almost linear between baseline and after cryopreservation. On the other hand we found that the concentration of MMP-9 in tissue sample extracts increased during storage with tumor samples showing the greatest change. Despite this, correlation analysis comparing MMP-9 levels at the beginning and end of cryopreservation was good according to Spearman rho.

The increased MMP-9 concentration in our tissue samples is difficult to explain. It might be the tissue homogenization process that is the culprit. In the process of homogenization, there is damage to the cell in the tissue samples and possible damage to the cells releases intracellular MMP-9 in the same way as in the coagulation process in serum samples [19]. In the method used in this study, we homogenized tissue samples and gently extracted supernatant after centrifugation providing aliquots for long-term cryostorage. The degree to which this process affects the level of MMP-9, however, is not known, and it might cause further leakage of MMP [27].

Although this might not fully explain the changes in concentration of MMP-9 in our tissue samples, it is possible that storage at − 80 °C is not adequate for tissue samples regarding analysis of MMP-9. This needs further research.

Our results concur with Tarr et al [23] who showed concentrations of MMP-9 in plasma samples to be stable at least 3 years at − 80 °C. Souza-Tarla et al. [22] reported MMP-9 levels to be stable at − 20 °C despite several thaw/freeze cycles, suggesting that MMP-9 is relatively stable. Our results do not concur with previous findings by Rouy et al. [17] who found that MMP-9 concentration in plasma drops dramatically during storage. However, the method used by Rouy et al. differed from ours in that they used pooled samples from different periods in time; we used different aliquots taken from the same plasma samples. This enabled us to follow variations at both individual and group levels.

The present study has some limitations: Samples were not consistent in terms of storage time; the period between sampling and baseline analysis varied since we analyzed specimens in batches. The time period between baseline analyses and postcryopreservation analyses differed between citrated plasma and tissue extract samples (9 and 12 years, respectively).

Numerous studies have used frozen blood and tissue samples to demonstrate the relationship between matrix metalloproteinase concentrations and colorectal cancer [2, 28, 29]. Plasma MMP-9 levels are significantly raised in patients with colorectal cancer and also decrease after tumor resection, suggesting MMP’s potential as a prognostic and diagnostic biomarker [11]. The demand for biomarkers has increased in modern healthcare as a result of our striving to achieve earlier diagnoses and tailored treatment for all patients. This study can be seen as a validation of MMP-9 as a biomarker of colon cancer, even in frozen citrated plasma that has been in long-term storage.

We have shown that MMP-9 is stable in citrated plasma samples kept frozen at − 80 °C for periods up to 9 years. This was not the case with supernatant extracts of tumor and tumor-free intestinal mucosa where levels increased over 12 years of storage. This is important information for further investigations using plasma MMP-9 level as a biomarker in CRC research.
